# Treatment Outcomes of Tocilizumab in Critically-Ill COVID-19 Patients, Single-Centre Retrospective Study

**DOI:** 10.3390/antibiotics11020241

**Published:** 2022-02-12

**Authors:** Wael Hafez, Mohamad Azzam Ziade, Arun Arya, Husam Saleh, Mahmoud Abdelshakor, Osman Fadl Alla, Pragati Agrawal, Sara Ali, Srinivasa Raghu Rao, Subrata Gupta, Ikram Abdelli, Honeymol Sebastian, Mohamed Ali, Muneir Gador, Ziad Al Baha, Ahmed Abdelrahman

**Affiliations:** 1 NMC Royal Hospital, Abu Dhabi P.O. BOX 35233, United Arab Emirates; azzamziade1@icloud.com (M.A.Z.); arun.arya@nmc.ae (A.A.); husam.saleh@nmc.ae (H.S.); mohamed.mahmoud@nmc.ae (M.A.); osman.blal@nmc.ae (O.F.A.); pragati.agrawal@nmc.ae (P.A.); sara.ali@nmc.ae (S.A.); srinivasa.rao@nmc.ae (S.R.R.); subrata.gupta@nmc.ae (S.G.); ikram.abdelli@nmc.ae (I.A.); honeymol.sebastian@nmc.ae (H.S.); mohamed.ali@nmc.ae (M.A.); muneir.gador@nmc.ae (M.G.); ziad.albaha@nmc.ae (Z.A.B.); ahmed.abdelrahman@nmc.ae (A.A.); 2 The Medical Research Division, Department of Internal Medicine, The National Research Center, Cairo 12622, Egypt; 3 Internal Medicine Department, Zagazig School of Medicine, Zagazig 44519, Egypt

**Keywords:** Tocilizumab, Interlukin-6, thrombosis, COVID-19, SARS-CoV-2, inflammation, D-Dimer

## Abstract

(1) Background: Severe COVID-19 outcomes are associated with cytokine release syndrome, characterized by the release of several immune modulators, including Interleukin-6 (IL-6). Tocilizumab (TCZ) is an IL-6 receptor antagonist used to treat rheumatic arthritis. The study aimed to evaluate the efficacy and safety of TCZ against COVID-19. (2) Methods: This was a retrospective study including 49 severe COVID-19 patients who received TCZ therapy in NMC Royal Hospital, UAE. (3) Results: Before Tocilizumab administration, the median temperature was 37.0 (IQR 36.0–39.6), and after day seven, the median reduced to 36.5 (IQR 35.8–37.9), *p* > 0.001. Thirty (61.2%) patients were admitted to the ICU, of which, eight (16.3%) were on WHO scale 4, sixteen (32.6%) on scale 5, and six (20.0%) on scale 6. TCZ reduced inflammatory markers over time, including CRP, D-Dimer, Ferritin, and Fibrinogen. By the end of week seven, 14 patients died (28.6%) while 35 (71.4%) improved and were discharged. (4) Conclusions: The study showed limited improvements in COVID-19 outcomes with TCZ therapy and highlighted the importance of D-Dimer monitoring for possible risk of thrombosis. Additionally, it could be recommended to upgrade the anti-coagulation dose to therapeutic levels once TCZ therapy is decided upon.

## 1. Introduction

Coronavirus disease 2019 (COVID-19) is caused by the severe acute respiratory syndrome coronavirus 2 (SARS-CoV-2) that arose in Wuhan (Hubei, China) in December 2019 [1]. The virus spread across the globe, causing a pandemic; as of 7 July 2021 it had affected more than 18 million, and killed over 4 million, people. Clinical presentation of COVID-19 ranges from being asymptomatic to multi-organ failure and death [2].

Cytokine Release Syndrome (CRS), mediated by viral entry into host cells, is more likely the cause of disease severity, unmet treatment outcomes, and high fatality rates. It is characterized by an uncontrolled immune response, over-activated macrophages and T-cells, and the release of several pro-inflammatory agents involving interleukin IL-1β (IL-1β), IL-6, tumor necrosis factor-α (TNFα), and interferon-γ (IFN-γ) [3,4].

CRS activation is associated with several life-threatening complications involving coagulopathy and multi-organ failure [5,6]. Studies that identified COVID-19 predictors of severe illness and high mortality rates reported elevated IL-6 levels among deceased cases compared to survivors [7].

The same is copied in many other studies, which concluded mounting levels of TNFα, IL-6, and IL-10 in severe COVID-19 patients in comparison to those with mild-to-moderate manifestations [2,8,9,10]. IL-6 is a major pro-inflammatory agent; it regulates the immune response to stress events such as injuries, malignancies, and viral infections. It stimulates the proliferation and differentiation of B-lymphocytes, T-helper, and natural killer (NK) cells [11,12].

IL-6 function is mediated by its binding to the interleukin-6 receptor (IL-6R) that presents in transmembrane and soluble forms. The next step is the activation of intracellular transcription pathways, including transmembrane glycoprotein 130 (gp130) and the Janus kinase/signal transducer and activator of transcription (JAK-STAT) pathway [13].

Cells lacking the transmembrane IL-6R cannot respond to IL-6, since gp130 alone has a low affinity for IL-6. Interestingly, soluble IL-6R (sIL-6R) can bind IL-6 with a similar affinity as the transmembrane IL-6R. Then, the IL-6–sIL-6R complex can bind to gp130 on cells, unresponsive to IL-6. This process is termed trans-signaling, and it permits IL-6 to modulate a wide spectrum of target cells [14]. It is believed that the classical IL-6 signaling pathway is anti-inflammatory, whereas the trans-signaling pathway is pro-inflammatory; however, the trans-signaling pathway could play a protective role in preventing tissue damage under certain conditions [15].

Embroilment of IL-6 in the fibrosis process by stimulating fibroblasts’ production and the release of procollagen and fibronectin explains the consequent lung cell damage caused by the exaggerated IL-6 release following SARS-CoV-2 infection [16].

Unwarranted IL-6–IL-6R signaling can also upregulate the expression of vascular endothelial growth factor (VEGF) and naïve T-cells’ maturation, as well as increase vascular permeability resulting in multi-organ damage [17]. Elevated sIL-6R during inflammatory reactions and infections stimulates different cells that are not normal targets for IL-6 [18,19].

Tocilizumab (TCZ) is a recombinant humanized monoclonal antibody, an FDA- approved IL-6 receptor antagonist for the treatment of rheumatic arthritis and severe CRS caused by chimeric antigen receptor T-cell (CAR-T) immunotherapy [20,21,22]. It targets both transmembrane and sIL-6R forms [23,24]. Although TCZ repurposing for CRS management among COVID-19 patients has been proposed, the effectiveness of TCZ for COVID-19 management remains uncertain due to controversial findings throughout recent studies.

Xu and colleagues investigated the efficacy of TCZ in the treatment of severe and critically-ill COVID-19 patients. They demonstrated that a single dose of TCZ improved the clinical symptoms immediately, reduced oxygen intake in 75% of patients, improved CT lung opacities in 90% of the patients, and promoted early discharges [25]. On the other hand, Campochiaro and fellows found no significant difference in clinical consequences between patients who received TCZ compared to standard treatment [26]. However, both studies included a small sample size, and Xu et al.’s study lacked a comparison group, which precluded clear conclusions on the efficacy of TCZ in severe COVID-19 patients.

The objective of this study was to examine the clinical findings’ efficacy and assess the short-term safety profile of TCZ therapy among critically-ill COVID-19 patients.

## 2. Results

### 2.1. Demographic and Clinical Characteristics of the Study Population

The study included 49 patients who received TCZ. The median age was 47.0 (IQR 16.0–89.0), male gender predominated (M/F: 41/8), and Asians made up the majority (31) of the patients (63.3%). Underlying comorbidities were hypertension (18%), diabetes (18%), cardiovascular disease (9%), and obesity (51%). The median time to viral clearance was 20 (IQR 3–60) days (Table 1).

In addition to TCZ, all patients received the standard treatments according to the UAE National guidelines for COVID-19 management, as displayed in Table 2. No significant difference was observed among different treatment groups regarding disease outcomes.

**Figure 1:**
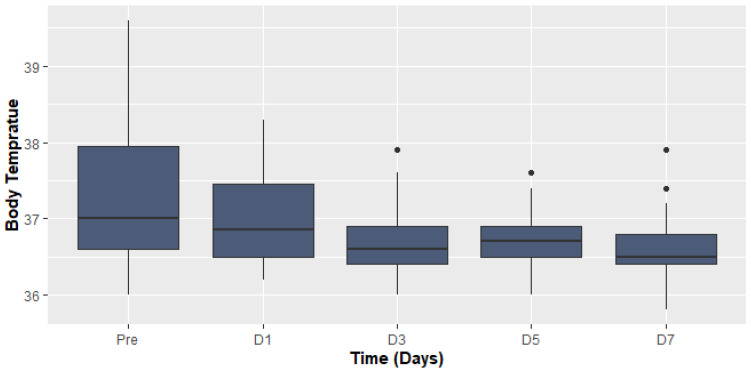
Body temperature before and after Tocilizumab.

The most common symptoms on admission were fever and dyspnea. The median body temperature before and after TCZ administration was (37.0 (IQR 36.0–39.6), 36.5 (IQR 35.8–37.9), *p* > 0.001, respectively) (Figure 1).

**Figure 2:**
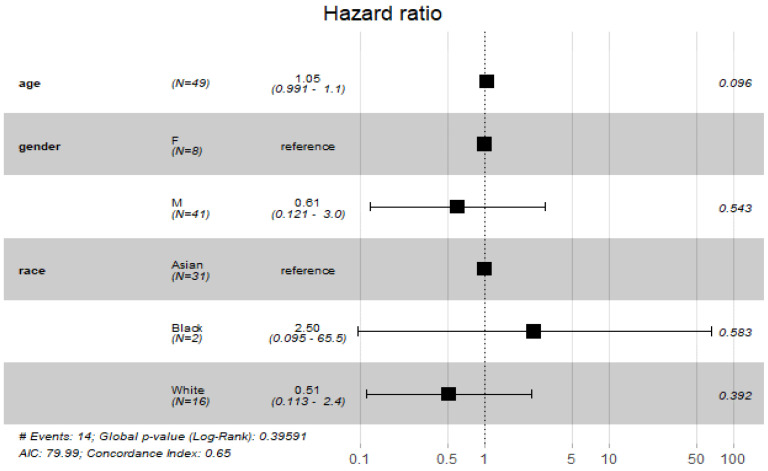
Cox proportional hazard model with demographic characteristics.

The findings of the COX proportional hazard regression model showed that patients’ demographics were not associated with a significant increase in odds of death among COVID-19 patients (Figure 2).

### 2.2. Classification of Patients before and after Treatment according to WHO Scale

Before TCZ administration, 30 (61.2%) patients were admitted to the ICU and 19 (39.8%) to the general ward. Eighteen (94.7%) patients were in the general ward, and eight (26.7%) patients admitted to ICU were on Scale 4. Among seventeen patients on Scale 5, one (5.3%) patient was in the general ward, and sixteen (53.3%) were in ICU, while all six patients on Scale 6 were ICU admitted patients.

In addition, 24 (54.5%) patients suffered from Low Flow Oxygen on Scale 4. Of patients with respiratory failure who needed non-invasive ventilation, there were about seventeen (34.7%) who were on Scale 5, and about six (12.2%) needed invasive ventilation with WHO Scale 6.

**Figure 3:**
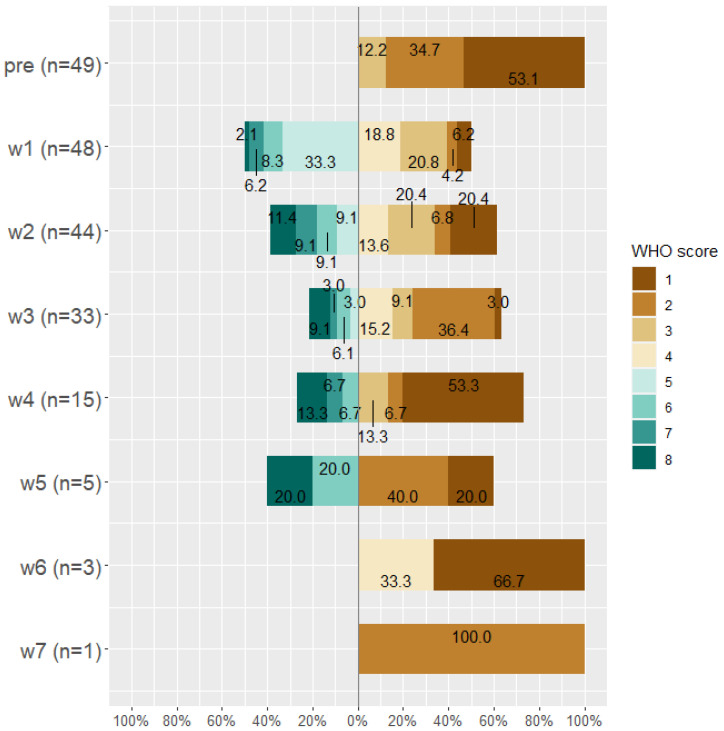
WHO score for all patients (*n* = 49) before taking Tocilizumab and at weeks 1, 2, 3, 4, 5, 6, and 7 after Tocilizumab administration.

**Figure 4:**
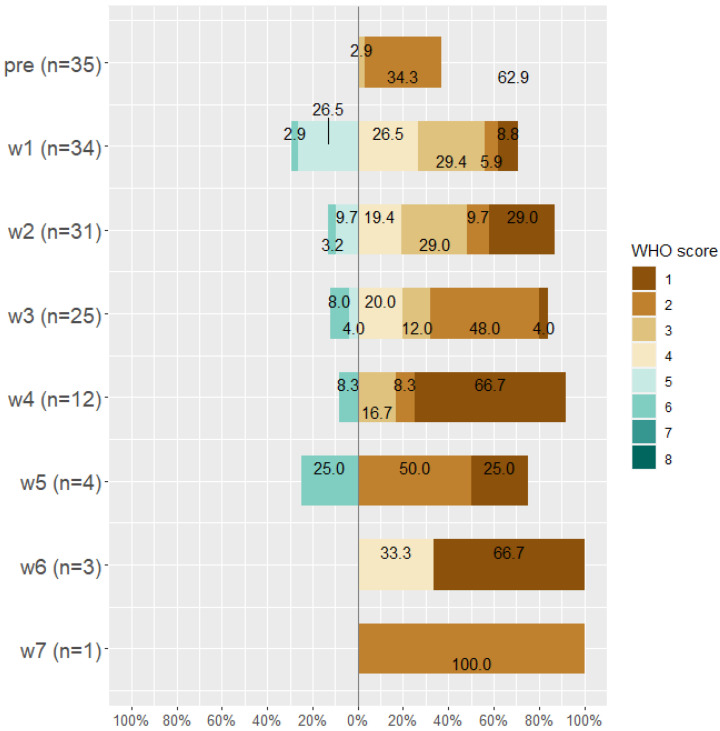
WHO score for improved patients (*n* = 35) before taking Tocilizumab and at weeks 1, 2, 3, 4, 5, 6, and 7 after Tocilizumab administration.

**Figure 5:**
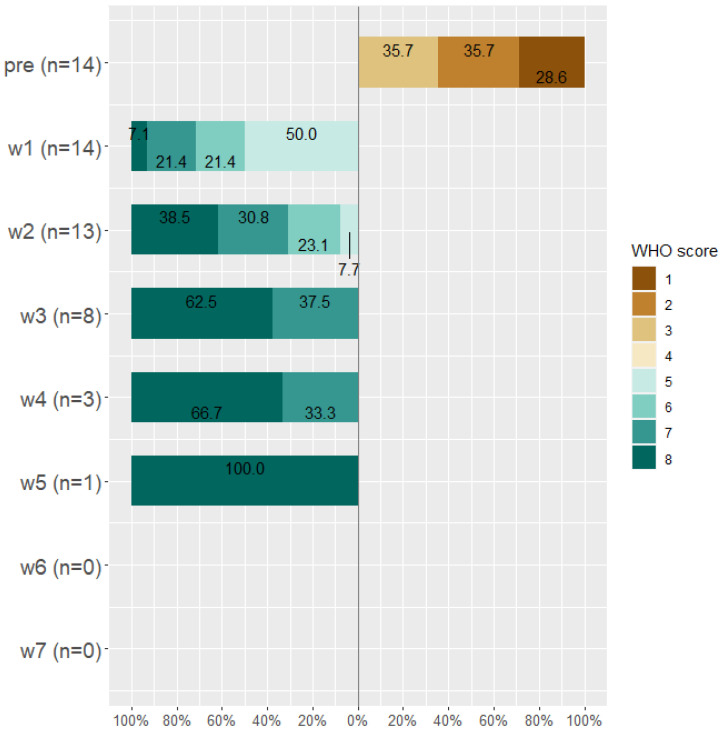
WHO score for worsened patients (*n* = 14) before taking Tocilizumab and at weeks 1, 2, 3, 4, 5, 6, and 7 after Tocilizumab administration.

By the end of week seven, about 14 patients died (28.6%), 19 (38.8%) patients were discharged from the hospital, 15 (30.6%) were discharged from the ICU, and the only remaining patient (0.02%) was on WHO Scale 2 and then discharged. Classification of patients according to WHO scale before and during Tocilizumab is displayed in Figure 3, Figure 4 and Figure 5.

### 2.3. Laboratory Findings before and after Tocilizumab Therapy

The laboratory findings of all patients on the day of TCZ administration until day seven among improved and deceased cases are summarized in Table 3. The Kruskal–Wallis test was used to analyze the change in the laboratory values over time and because of the small sample size. However, two patients were excluded from the analysis because they were sent to another hospital after three days of infection. The change in laboratory findings over time among improved patients is shown in Table 4, and among worsened patients in Table 5.

**Figure 6:**
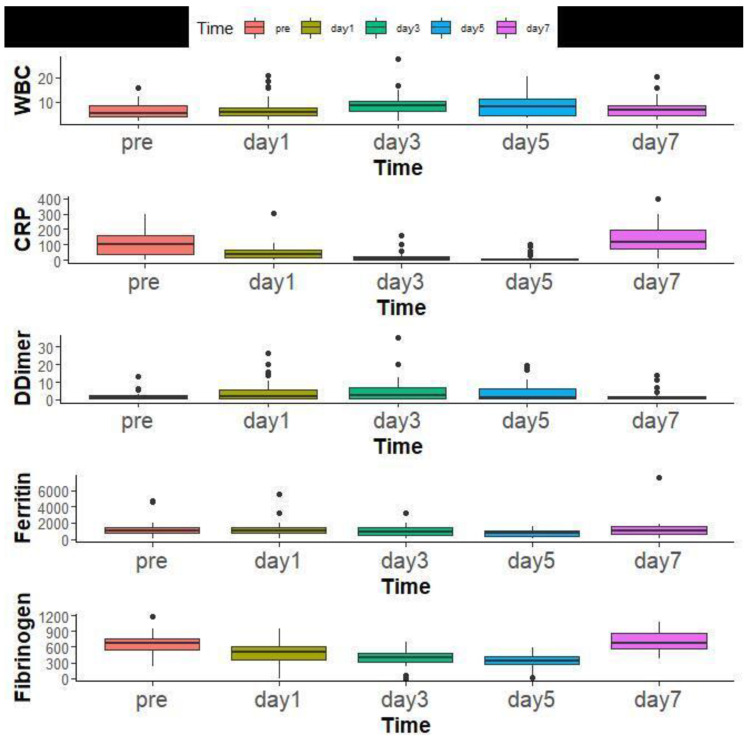
Laboratory findings of all patients overtime before Tocilizumab administration and at days 1, 3, 5, and 7.

The median WBCs Count before TCZ administration was 6.79 (IQR 2.40–20.3) and significantly increased over time. The inflammatory markers, including CRP, D-Dimer, Ferritin, and Fibrinogen, also significantly improved over time (Figure 6).

**Figure 7:**
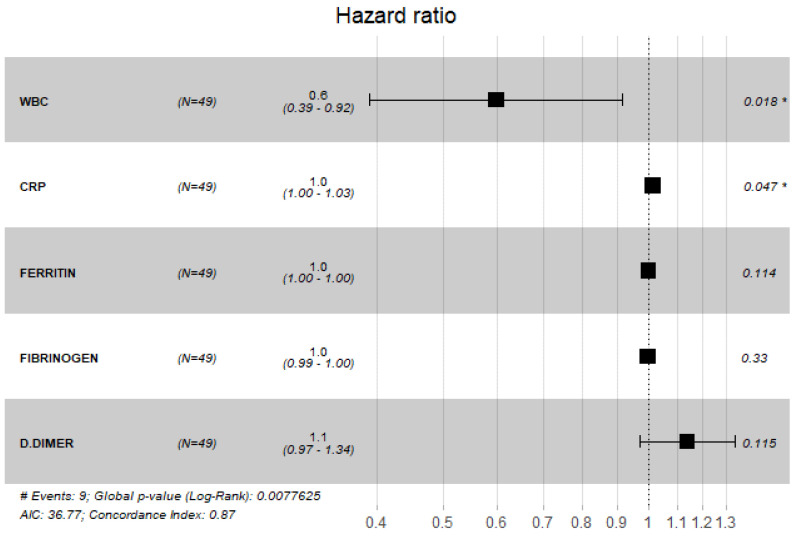
Cox proportional hazard model with laboratory values.

The findings of COX proportional hazard regression model revealed a significant increase in the odds of mortality with elevated WBC count and CRP ((RR = 0.60, 95% CI: (0.39, 0.92), *p* = 0.018), (RR = 1.00, 95% CI: (1.00, 1.03), *p* = 0.047), respectively) (Figure 7).

### 2.4. Kaplan–Meier Survival Curves after Tocilizumab Therapy

**Figure 8:**
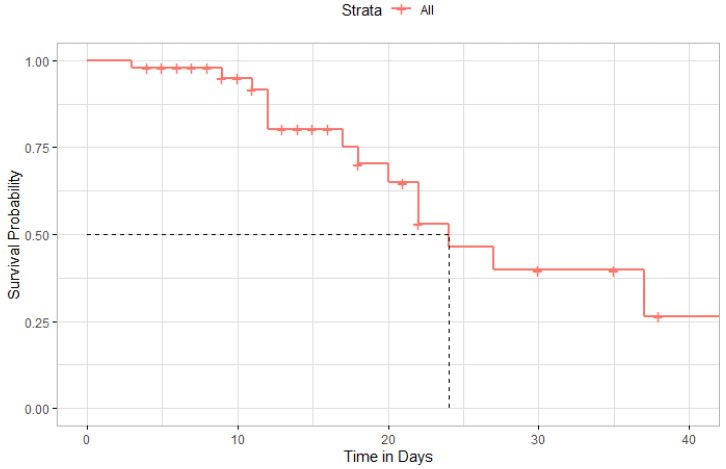
Kaplan–Meier Curve of survival rate after administration of Tocilizumab.

**Figure 9:**
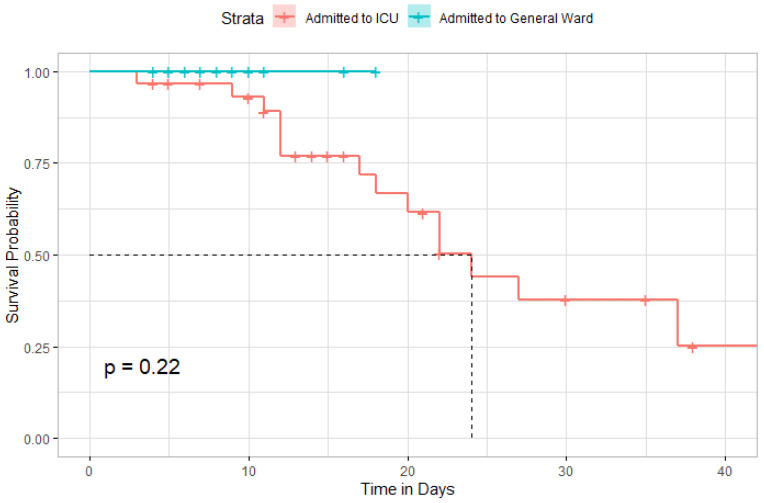
Kaplan–Meier curve comparing the survival distribution between admitted patients to ICU or general ward.

Figure 8 depicted the Kaplan–Meier curve after administration of TCZ among all patients included in the study. The median time to survival among included patients was 24 days (95% CI, 20-Inf). However, the difference in survival rates was not statistically significant among patients admitted to the ICU compared to those admitted to the general ward (Figure 9).

### 2.5. The Time until Viral Clearance between ICU Patients and General Ward

**Figure 10:**
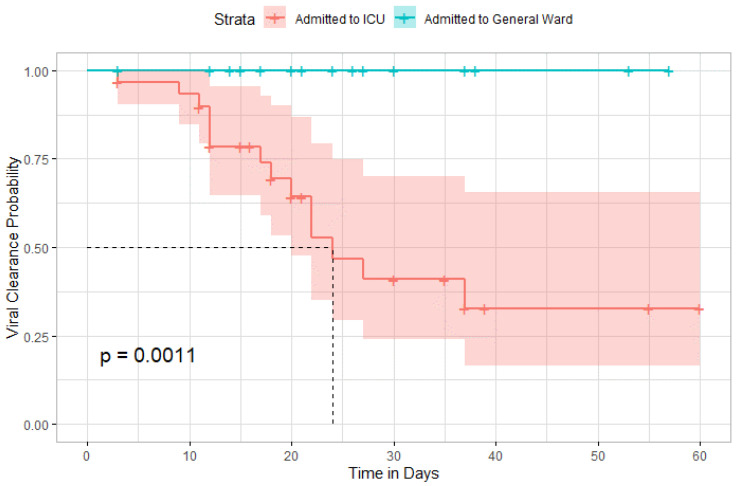
Kaplan–Meier curve comparing the time until viral clearance between ICU admitted patients and general ward.

Time until viral clearance was significantly different among patients in the ICU and those in the general ward (*p* = 0.001) (Figure 10).

### 2.6. Safety Findings

In our study, we observed secondary bacterial and fungal infections, rapid worsening desaturation, and elevated liver function tests. Secondary infections were observed among patients with late onset of TCZ therapy.

## 3. Discussion

This was an observational study of critically-ill COVID-19 patients who received TCZ. Before treatment initiation, 30 (61.2%) patients were admitted to the ICU. At week seven post-TCZ, 30.6% of the patients were discharged from the ICU, 38.8% were discharged from the hospital, 28.6% were deceased, and only 0.02% remained in the medical ward and were then discharged later.

Luo et al.’s study was among the first observational studies evaluating the efficacy of TCZ therapy among COVID-19 patients. They reported a positive role of TCZ in CRS management, but multiple doses might be required for critically ill patients [27].

Guaraldi et al. observed that TCZ was associated with decreased mechanical ventilation and mortality among patients with COVID-19 associated pneumonia [28]. Their findings were consistent with Klopfenstein et al.’s study [29].

Another randomized, open-label trial reported that TCZ decreased invasive and non-invasive ventilation requirement and mortality rates at day 14, but some of the patients in TCZ treatment groups received dexamethasone; this could interfere with our interpretation of the efficacy of TCZ [30].

Campochiaro et al. compared COVID-19 patients on high-flow supplemental oxygen and receiving TCZ and those receiving standard treatments; they found no statistical significance regarding clinical improvement and mortality between both groups during 28-day follow-up [26]. These findings were also observed by Salvarani et al., who reported no benefit of TCZ on disease progression compared to standard care. However, several patients in the control group received TCZ as rescue therapy; this could interfere with the interpretation of the influence of TCZ on the clinical outcomes [31].

In our study, inflammatory markers were improved after initiation of TCZ and CRP levels improved at day seven and returned to the normal levels; this finding was also observed by Guaraldi et al. [28]. Other inflammatory markers, including fibrinogen and ferritin, were also significantly improved in our study; this finding was also reported in different studies evaluating the efficacy of TCZ [32,33,34], but our observation regarding D-Dimers levels was distinct from that of Corominas, as we observed increased levels of D-Dimer during the first week of the treatment period. Elevated D-Dimer, CRP levels, and arterial and venous thromboembolism were observed in a case series of severe COVID-19 patients, indicating that TCZ could mask the inflammatory markers while not decreasing the risk of thrombotic events [35].

Xu et al. reported that TCZ is effective for managing COVID-19; it decreased oxygen requirement in 75% of their cohort, lung opacities were improved by 90.5%, and CRP levels and lymphocytic counts returned to normal values within five days. However, the study has several limitations, such as small sample size and high risk of bias; the study was also a single-arm study in one center with a short observation period [25].

The interplay between IL-6, IL-6 receptor antagonists, and venous thromboembolism is complex. IL-6 was reported to contribute to deep vein thrombosis via induction of hepatic thrombopoietin and dysregulation of miR-338–5p expression [36,37]. On the other hand, IL-6 suppression and antagonism were reported to increase thrombus formation and reduce factor XIII, chimerin, and plasminogen activator inhibitor, leading to thrombus instability and increased risk of thrombosis [38,39]. Thus, IL-6 seems to be involved in thrombus formation and resolution.

In our study, two patients developed rapid progressive desaturation and respiratory failure following TCZ and died, though the primary cause of death was not determined; in all cases, there was insufficient time to perform CT pulmonary angiography. However, it was noted that both patients had a sharp rise in D-Dimer following TCZ; additionally, they were on prophylactic dose low-molecular-weight heparin. Therefore, we decided to upgrade the dose to therapeutic levels once it was decided to initiate TCZ. This practice improved the outcomes, with no further worsening following TCZ.

The use of a therapeutic dose or a prophylactic dose of heparin is considered an area of debate nowadays. A therapeutic dose of heparin was shown to increase the probability of survival and decrease the need for organ support compared to the prophylaxis doses among non-critically ill COVID-19 patients [40]. It also decreased the risk of mortality and thromboembolism among patients with elevated D-Dimer in the HEP-COVID multicenter randomized clinical trial [41]. However, the therapeutic dose of heparin showed no significant effect compared to the prophylactic dose of heparin in decreasing ICU admission or the need for mechanical ventilation among moderately ill COVID-19 patients. Still, it was associated with decreased odds of mortality [42]. Furthermore, the use of a therapeutic dose of the direct oral anticoagulants was not recommended based on the “ACTION” clinical trial [43]. Moreover, therapeutic doses of heparin did not increase the chance of survival or the need for organ support compared to the prophylactic doses among critically ill COVID-19 patients [44]. Based on these findings, it is worth mentioning that the protective effect of the anti-coagulation therapies is controlled by the initiation time in relation to the degree of hyper-inflammation and coagulation status. However, the use of therapeutic doses of anticoagulants appeared to be beneficial in our study.

A large cohort study of ICU-admitted COVID-19 patients reported a decline in mortality rates when TCZ was administered within the first two days of ICU admission, indicating that early initiation of TCZ could be more effective and life-saving [45]. A similar observation was reported by Sciascia et al. [32].

There are several safety concerns surrounding COVID-19’s treatment with TCZ, including higher bacterial and fungal infections [26,28,46,47]. Still, the risk of secondary infection could be attributed to the fact that most of these studies included critically ill patients in the ICU, which is associated with higher secondary infection rates [48,49]. Other reported safety concerns include hepatotoxicity, intestinal perforation, hypertriglyceridemia, and thrombocytopenia [50,51].

The mechanism of action of TCZ depends on its inhibitory effect of IL-6R, therefore decreasing IL-6 mediated increased vascular permeability, immune cells activation, histamine release from mast cells, activation of coagulation, and development of disseminated intravascular coagulation (DIC) [52]. Abnormal IL-6 production is mediated by SARS-CoV-2 binding to angiotensin-converting enzyme-2 (ACE-2) receptor, internalization to the host cells [53], and ACE-2 shedding by disintegrin and metalloproteinase domain 17 (ADAM17); it was reported that binding of the virus to ACE-2 upgrades ADAM17 and exaggerates its function [54]. ADAM17 mediates the release of active forms of pro-inflammatory agents, including IL-6 and TNFα [55].

According to a virus–cell interaction study, binding of the SARS-CoV-2 spike protein leads to the upregulation of angiotensin II type 1 receptor (AT1), upregulates ADAM17, and increases the release of sIL-6R and initiation of the hyper-inflammatory response. The authors confirmed their findings by using Candesartan cilexetil, an AT1 receptor antagonist, which resulted in down-regulation of IL-6–sIL-6R release in spike-expressing cells [56].

This mechanism could explain the contribution of SARS-CoV-2 entry and pathogenesis of CRS, IL-6 release, and that the potential role of TCZ in the management of COVID-19 could be mediated through its inhibitory effect on sIL-6R.

Limitations of our study include the nature of the retrospective-observational study. We could not draw a clear, definite conclusion regarding the efficacy and safety of TCZ in the absence of a control group. Further, our study was conducted during the first wave of the pandemic, and there are five variants of SARS-CoV-2 identified so far: Alpha, Beta, Gamma, Delta, and Omicron. A recent systematic review showed that the Beta and Delta variants are associated with a higher risk of disease severity, mortality, and hospitalization rate than the wild-type virus and other variants [57]. Thus the effect of TCZ on the clinical outcomes of COVID-19 should be interpreted with caution due to the difference in the viral variants and its possible implications on disease outcomes.

## 4. Materials and Methods

### 4.1. Institutional Review Board IRB

This study was performed according to the Declaration of Helsinki. The study was reviewed and accepted by the NMC Central scientific committee (NMCHC/CR/AUH/CSC/APP/002), NMC Regional Research Ethics Committee (NMC/PREC/AUH/2020/0011), and Abu Dhabi Health COVID-19 Research Ethics Committee, Abu Dhabi, UAE Committee (DOH/CVDC/2020/2010).

### 4.2. Study Design and Study Population

This was a non-interventional retrospective study of *p* COVID-19 patients treated in NMC Royal Hospital, Khalifa City, Abu Dhabi, UAE between 1 April 2020 and 15 June 2020.

The study included hospitalized adult COVID-19 patients as confirmed by real-time polymerase chain reaction (RT-PCR) assay developed from the publicly released virus sequence using nasopharyngeal swabs under aseptic conditions.

Inclusion criteria were hospitalized adult patients (≥18 years) with COVID-19, proven by clinical and radiological signs of possessing progressive disease, in addition to laboratory evidence indicative of cytokine storm complications diagnosed prior to or at the time of admission. Clinical signs included fever ≥38.5 °C, dyspnea or respiratory rate (RR) >30 breaths/min, and/or oxygen saturation SPO_2_ <93% in room air or increased oxygen therapy requirement to maintain SPO_2_ >94%. Radiological findings included CXR and/or CT suggesting pneumonia and one or more laboratory findings suggesting cytokine storm, such as IL-6 >3, ferritin >300 ug/L with doubling within 24 h, or ferritin >600 µg/L at admission, LDH >250 µ/L, and elevated D-Dimer >1 ng/mL.

Patients who were pregnant, with active tuberculosis, with allergy to TCZ or other monoclonal antibodies, who were treated previously with other immunomodulatory drugs (including TCZ), with elevated AST/ALT >5 times, or with platelet counts <50,000 (×10^3^/µL) or neutrophil counts <500 (×10^3^/µL) were excluded.

### 4.3. Treatment

All patients received standard care following the UAE national guidelines for COVID-19 management. Antiviral therapy included hydroxychloroquine (HCQ) plus favipiravir, HCQ plus lopinavir/ritonavir, or HCQ plus both favipiravir and lopinavir/ritonavir.

The dose of favipiravir was 1600 mg BID for one day followed by 600 TID for a total of seven days. Lopinavir/ritonavir doses were two tablets 200/50 mg BID not exceeding ten days, and that of HCQ was 400 mg BID for two doses then 200 mg BID for a total of seven days. Tocilizumab was administered via intravenous drip-up over 1 h, with a loading dose of 4–8 mg/kg body weight, and the recommended dose was 400 mg to a maximum of 800 mg. An additional dose of Tocilizumab was given in case of persistent fever and/or hypoxia within 12–24 h, except for one patient, who received three doses. Tocilizumab was first used in patients who did not respond or deteriorated clinically after receiving steroidal therapy (methylprednisolone) at a dose of 40 mg every 8 h for three days.

### 4.4. Data Collection

Before and after TCZ therapy, baseline laboratory tests were performed, including a complete blood count, C-reactive protein (CRP), D-Dimer, lactate dehydrogenase (LDH), liver function tests, kidney function tests, lymphocytic count, fibrinogen, and serum ferritin.

All patients had chest X-ray and/or CT upon presentation and follow-up chest X-ray and/or CT after seven days of treatment with TCZ. SPO_2_ and oxygen requirements were measured before and after TCZ treatment and were followed up for seven days or more as needed.

### 4.5. Study Outcomes

The main goal of the study was to assess the clinical outcome of TCZ treatment from week one to week seven in patients with severe COVID-19 using the WHO 8 ordinal scale for clinical improvement. A positive outcome is associated with two points of improvement on the scale.

Worsened or deceased patients were defined as those who worsened then died after Tocilizumab, while improved or stable patients were those who improved after Tocilizumab and were then discharged.

The efficacy and safety of Tocilizumab therapy in severe COVID-19 patients were evaluated as secondary study outcomes, short-term safety measurements within 28 days (to exclude the confounding effect of severe immunocompromization and severe secondary bacterial infections on outcomes) including the occurrence of adverse drug reactions, elevated transaminase, neutropenia, secondary bacterial infections, and significant gastrointestinal symptoms. Frequency of patients who needed invasive ventilation, intensive care unit (ICU) admission, and ICU mortality were also measured.

### 4.6. Statistical Analysis

Continuous variables are described as median and interquartile range (IQR) and they are compared by Wilcoxon rank sum test. Categorical variables are described as numbers and percentages and they are compared by using Fischer exact test due to small sample size. Clinical status was assessed by the WHO 8 ordinal scale and they were described as number (%) before taking TCZ and at weeks 1, 2, 3, 4, 5, 6, and 7 after Tocilizumab administration. Changes in clinical and biochemical variables before and after TCZ treatment were evaluated at fixed time points (days 3–7) post-Tocilizumab. Survival analysis was performed using the Kaplan–Meier approach, and comparisons were made using the Log Rank Test. The COX Proportional Hazard Regression Model was conducted to investigate factors influencing the risk of death among COVID-19 patients. After data collection and verification, all data were entered for statistical analysis using R Software version 3.5.2 (20 December 2018) “Eggshell Igloo”. The confidence interval was set to 95% and the *p*-value (*p* < 0.05) was considered significant (S) and *p* < 0.01: Highly significant (HS).

## 5. Conclusions

Tocilizumab is a potential therapeutic agent for severe and critically-ill COVID-19 patients; it resulted in clinical improvement of patients, reducing levels of several inflammatory markers including CRP, LDH, and fibrinogen. However, we observed and highlighted the importance of D-Dimer monitoring for possible risk of thrombosis; furthermore, it could be advisable to upgrade the anti-coagulation dose to therapeutic levels once TCZ therapy is decided upon. More research investigating the factors influencing the efficacy and safety of TCZ is needed.

## Figures and Tables

**Table 1:** Clinical characteristics of the patients.

Characteristics	Overall (*n* = 49)	Improved or Stable (*n* = 35) (1)	Worsened or Deceased (*n* = 14) (1)	*p*-Value (2)
Age	47 (41, 58)	45 (40, 54)	60 (47, 66)	0.013
SEX				0.7
*Female*	8 (16%)	5 (14%)	3 (21%)	
*Male*	41 (84%)	30 (86%)	11 (79%)	
Race/ethnicity				0.3
*Asian*	31 (63%)	24 (69%)	7 (50%)	
*Black*	2 (4.1%)	1 (2.9%)	1 (7.1%)	
*White*	16 (33%)	10 (29%)	6 (43%)	
Hypertension				0.031
*NIL*	4 (8.2%)	3 (8.6%)	1 (7.1%)	
*No*	27 (55%)	23 (66%)	4 (29%)	
*Yes*	18 (37%)	9 (26%)	9 (64%)	
Diabetes Mellitus				0.5
*NIL*	4 (8.2%)	3 (8.6%)	1 (7.1%)	
*No*	27 (55%)	21 (60%)	6 (43%)	
*Yes*	18 (37%)	11 (31%)	7 (50%)	
Cardiovascular Diseases				0.4
*NIL*	6 (12%)	3 (8.6%)	3 (21%)	
*No*	34 (69%)	26 (74%)	8 (57%)	
*Yes*	9 (18%)	6 (17%)	3 (21%)	
BMI	25 (13, 33)	25 (12, 34)	24 (16, 28)	0.6
WHO scale before receiving Tocilizumab				0.005
*4*	26 (53%)	22 (63%)	4 (29%)	
*5*	17 (35%)	12 (34%)	5 (36%)	
*6*	6 (12%)	1 (2.9%)	5 (36%)	
Time to viral Clearance (from first positive till first negative)	20 (12, 27)	20 (15, 32)	18 (12, 22)	0.2

(1) Median (IQR); *n* (%); (2) Wilcoxon rank-sum test; Fisher’s exact test.

**Table 2:** Drugs taken by the study population.

Drugs Taken by Patients	Overall (*n* = 49) (1)	Improved or Stable (*n* = 35) (1)	Worsened or Deceased (*n* = 14) (1)	*p*-Value (2)
HCQ Azithromycin	4 (8%)	4 (11.5%)	0 (0%)	0.3
HCQ Favipiravir	23 (47%)	16 (46%)	7 (50%)	0.9
HCQ. Azithromycin Favipiravir	11 (22%)	7 (20%)	4 (28.5%)	>0.9
HCQ Favipiravir Lopinavir/Ritonavir	6 (12%)	4 (11.5%)	2 (14%)	>0.9
HCQ. Favipiravir Lopinavir/Ritonavir Azithromycin	4 (8%)	4 (11.5%)	0 (0%)	

(1) *n* (%); (2) Fisher’s exact test; Pearson’s Chi-squared test.

**Table 3:** Laboratory findings before and during Tocilizumab therapy among total population.

Laboratory Findings (Median, IQR)	Before Tocilizumab (*n* = 47)	Day 1 (*n* = 47)	Day 3 (*n* = 47)	Day 5 (*n* = 47)	Day 7 (*n* = 47)	Total	*p*-Value
Hemoglobin Level (g/L)	13.7 (9.00, 16.7)	13.2 (7.80, 16.1)	12.6 (8.20, 16.2)	12.7 (6.30, 15.7)	13.1 (7.30, 16.5)	13.2 (6.30, 16.7)	0.546
White Blood Cell Count (×10^9^/L)	6.79 (2.40, 20.3)	5.30 (2.10, 15.8)	5.47 (2.31, 20.8)	8.57 (2.21, 27.6)	8.60 (3.39, 20.9)	6.72 (2.10, 27.6)	0.007
Neutrophils (×10^9^/L)	75.1 (35.5, 95.0)	74.0 (24.6, 93.2)	69.7 (20.5, 92.3)	71.6 (31.4, 94.3)	63.1 (17.4, 94.6)	72.6 (17.4, 95.0)	0.599
Lymphocytes (×10^9^/L)	18.8 (2.20, 52.6)	18.4 (3.50, 65.2)	18.8 (4.60, 70.8)	16.9 (3.40, 58.2)	24.7 (3.50, 63.4)	18.8 (2.20, 70.8)	0.818
Neutrophil–Lymphocyte Ratio	4.10 (0.600, 43.1)	4.00 (0.300, 26.6)	3.60 (0.290, 19.5)	4.20 (0.540, 27.7)	2.50 (0.200, 27.0)	3.80 (0.200, 43.1)	0.801
C-Reactive Protein (mg/L)	120 (12.0, 400)	109 (2.00, 302)	39.0 (4.00, 302)	10.5 (0.310, 164)	4.50 (0.150, 102)	39.0 (0.150, 400)	<0.001
D-Dimer (ng/mL)	0.940 (0.140, 14.1)	1.15 (0.200, 13.5)	1.95 (0.150, 26.6)	2.49 (0.270, 35.2)	1.22 (0.100, 19.3)	1.21 (0.100, 35.2)	0.017
Ferritin (ng/mL)	1000 (120, 7620)	1080 (145, 4750)	1010 (74.4, 5520)	921 (73.6, 3180)	704 (49.8, 1650)	932 (49.8, 7620)	0.032
Lactate Dehydrogenase (U/L)	434 (194, 1390)	472 (271, 1080)	531 (207, 1550)	430 (270, 1720)	361 (83.0, 2530)	454 (83.0, 2530)	0.241
Fibrinogen (mg/dL)	664 (370, 1100)	667 (235, 1180)	514 (5.28, 973)	402 (3.40, 711)	334 (17.6, 636)	514 (3.40, 1180)	<0.001

**Table 4:** Laboratory findings before and during Tocilizumab therapy among improved patients.

Laboratory Findings for Improved Patients	Before Toci. (*n* = 34) (1)	Day 1 after Toci. (*n* = 34) (1)	Day 3 after Toci. (*n* = 34) (1)	Day 5 after Toci. (*n* = 34) (1)	Day 7 after Toci. (*n* = 34) (1)	*p*-Value (2)
Haemoglobin(g/L)	13.80 (12.30, 14.38)	13.20 (11.90, 14.00)	13.20 (12.05, 13.83)	13.70 (11.80, 14.10)	13.15 (12.07, 14.53)	0.8
White blood cells count (×10^9^/L)	6.8 (4.0, 8.1)	5.6 (3.9, 9.4)	5.7 (4.0, 10.0)	8.6 (6.0, 12.2)	7.3 (5.0, 10.6)	0.063
Neutrophils count (×10^9^/L)	74 (58, 84)	74 (63, 81)	68 (54, 80)	71 (57, 82)	60 (42, 80)	0.4
Lymphocytes Count (×10^9^/L)	20 (10, 35)	18 (12, 28)	20 (14, 32)	17 (11, 28)	29 (12, 44)	0.5
Neutrophil Lymphocyte ratio	3.7 (1.5, 8.3)	4.0 (2.3, 6.6)	3.5 (1.7, 5.5)	4.2 (2.0, 7.8)	2.0 (0.9, 7.1)	0.5
C-reactive protein (mg/L)	119 (70, 197)	106 (40, 146)	39 (17, 64)	13 (4, 23)	4 (4, 12)	<0.001
D-Dimer (ng/mL)	0.9 (0.5, 2.2)	1.1 (0.6, 2.0)	1.7 (0.8, 6.1)	1.8 (0.5, 4.3)	0.8 (0.3, 3.8)	0.088
Ferritin (ng/mL)	1000 (437, 1644)	996 (670, 1491)	950 (714, 1381)	891 (527, 1353)	639 (382, 945)	0.046
Lactate dehydrogenase (U/L)	420 (375, 512)	456 (383, 579)	505 (395, 593)	406 (340, 540)	309 (266, 583)	0.05
Fibrinogen (mg/dL)	644 (574, 833)	672 (526, 741)	514 (417, 595)	419 (319, 480)	347 (278, 453)	<0.001

(1) Median (IQR); (2) Kruskal–Wallis rank-sum test.

**Table 5:** Laboratory findings before and during Tocilizumab therapy among worsened patients.

Laboratory Findings for Worsened Cases	Before Toci (*n* = 13) (1)	Day 1 after Toci (*n* = 13) (1)	Day 3 after Toci (*n* = 13) (1)	Day 5 after Toci (*n* = 13) (1)	Day 7 after Toci (*n* = 13) (1)	*p*-Value (2)
Haemoglobin (g/L)	13.40 (12.50, 13.90)	12.50 (11.30, 13.70)	12.00 (11.23, 13.12)	12.55 (11.60, 13.75)	12.55 (10.83, 13.40)	0.6
White blood cells count (×10^9^/L)	6.8 (4.6, 9.9)	4.6 (4.2, 6.7)	5.4 (4.5, 6.7)	7.6 (4.0, 9.7)	9.6 (4.4, 13.2)	0.3
Neutrophils count (×10^9^/L)	78 (70, 86)	75 (65, 78)	78 (66, 81)	70 (54, 74)	69 (50, 86)	0.6
Lymphocytes Count (×10^9^/L)	15 (10, 24)	19 (16, 26)	16 (14, 26)	19 (14, 35)	21 (10, 34)	0.8
Neutrophils lymphocytes ratio	5.0 (2.8, 8.3)	4.0 (2.4, 4.7)	4.8 (2.5, 5.9)	3.5 (1.5, 5.5)	3.6 (1.5, 8.4)	0.8
C-reactive protein (mg/L)	125 (81, 191)	119 (74, 171)	39 (25, 58)	8 (4, 28)	4 (4, 14)	<0.001
D-Dimer (ng/mL)	1.0 (0.5, 1.3)	1.2 (0.6, 2.5)	2.5 (1.0, 5.3)	4.0 (0.9, 8.7)	2.2 (0.7, 12.2)	0.2
Ferritin (ng/mL)	858 (742, 1304)	1133 (863, 1469)	1128 (854, 1485)	938 (648, 1395)	872 (642, 1040)	0.4
Lactate dehydrogenase (U/L)	509 (455, 604)	550 (469, 810)	630 (411, 696)	538 (378, 802)	441 (319, 855)	>0.9
Fibrinogen (mg/dL)	671 (556, 860)	629 (564, 790)	544 (310, 639)	400 (275, 457)	294 (246, 356)	<0.001

(1) Median (IQR); (2) Kruskal–Wallis rank-sum test.

## Data Availability

Data can be available upon request from the first and corresponding author.
